# Psychiatrist and the science of criminology: Sociological, psychological and psychiatric analysis of the dark side

**DOI:** 10.4103/0019-5545.31511

**Published:** 2007

**Authors:** T. S. Sathyanarayana Rao

**Affiliations:** Department of Psychiatry, J. S. S. Medical College and Hospital, Ramanuja Road, Mysore - 570 004, Karnataka, India

Humans have dark side that loves crime and violence. We all may deny it but the contrary is true. And it applies regardless of our age, caste, social status, region, religion or education. The television, movies, sports and many happenings reported in news papers and magazines are indicative of our obsession with the fact and fiction of crime and violence. Modern culture is infact can be referred to as the most violent culture in history in the number of crimes and in the nature brutality.[1]

Recently, Nithari hogged the lime light and the press was full of wild speculations and assumptions.[2] It was so much everywhere because crime somehow intrigues people. It can attract or repel but it does happen. It can amuse so also frighten us. It can generate anger when it affects near or dear ones in our community. The crime arouses so much of interest or passion, yet its understanding as to why it occurs and what we can do about it has often remained a problem. Public officials, politicians, experts and consultants and anyone who matters often offer simple and incomplete discourses on the events and method of solution for eradicating crime. Solutions offered may be more policing, setting up of closed circuit TVs, increasing street lighting, putting up sturdy locks, karate classes for the people on the one hand and stiff penalties, speedy imprisonment or capital punishment on the other hand for the criminals. ‘Experts’ dole out abstract interpretations that have any practical value. In short, as in most areas of human behavior, there is no shortage of experts but there are few effective solutions.[3]

Bartol argues effectively that ‘our inability to prevent crime is partly due to our problems in understanding criminal behavior, a complex phenomenon. Since crime is complex, it goes without saying that explanations of crime require complicated, involved answers. Research indicates that most people have a very limited tolerance for complexity and ambiguity. They apparently want simple, straight forward answers for even a very complex issue. As behavioral scientists we need to understand that criminal behavior is a vastly complex, yet poorly understood phenomenon. There is no all encompassing psychological explanation for crime, than there is sociological, anthropological, psychiatric, economic or historic one.[3] Without the help of many disciplines, for sociology or psychology to reach the basic ‘truth’ is almost impossible. In most, understanding criminal behavior calls for an inter disciplinary approach integrating data, theory and the practical view point of each discipline.

## HUMAN NATURE AND CRIME

Bartol[3] again reviews underlying assumptions about human nature and identifies 3 major domains:

1. ‘Conformity’ perspective: Classical example is the strain theory of Merton R. K. which argues that ‘humans are fundamentally good people and conforming beings who are strongly influenced by values and attitudes of the society in which they live. It assumes that humans, as creatures of conformity who want to do the ‘right’ thing’. ‘Right’ thing therefore is what the society says is the ‘right’ thing. For example, the American society advocates ‘accumulation of wealth or status is all important and many continue to accept these well advertised goals’. Education, social network, contacts and family influence can help access these to many but not to all. When there is ‘perceived discrepancy’, between materialist values and goals cherished and the availability of legitimate means, then crime and delinquency occurs. Groups and individuals experience high level of ‘strain’ are forced to decide whether to accept of violate norms or laws, consequently they conform, withdraw or rebel.
2. ‘Non-conformist’ perspective: Assumes that human beings are basically undisciplined creatures, given the chance would flout society's convention and commit crime indiscriminately. Travis Hisschi's[3] social control theory contentes that crime and delinquency occur when an individuals ties to the conventional order or normative standards are weak or largely nonexistent, where checks and balances in the society are at fault. This assumes that human nature in fundamentally ‘bad’ or ‘antisocial’.
3. The third perspective assumes that human beings are basically ‘neutral’ by birth and they learn all their behaviors, beliefs and tendencies from social environment and this is the cornerstone of learning approach. A good example is Sutherland's differential association theory. Accordingly, criminal behavior is learned through social interaction with other people. “It is not the result of emotional disturbance, mental illness or innate qualities of ‘goodness’ or ‘badness’ “……. People learn to be criminal as a result of messages they get from others, who were also thought to be criminals. The conventional wisdom that “bad company promotes bad behavior” aptly summarizes the theme.

## ROLE IN CRIMINOLOGY

Hence, criminology utilizes multi-disciplinary approach. Knowledge about criminal actions encompasses psychology, sociology, psychiatry, anthropology, biology, neurology, political science, economics etc. Though our expertise is in the field of psychological principles, we need to learn concepts, theories and research knowledge from other disciplines to understand, explain and prevent criminal behavior.

## SOCIOLOGICAL ISSUES

The demographic and group variables such as “age, race, gender, socio-economic status, interpersonal relationships, ethnic-cultural application” are significant for certain categories and patterns of crime. It also probes situational or environmental factors that are conducive to criminal action such as the time, place, kind of weapon used, circumstances surrounding the crime etc. In the broader sense, it covers underlying social conditions - which may be inequities in education, employment or criminal justice system.

## PSYCHOLOGICAL ISSUES

What can be called ‘Psychological criminology’ encompasses science of behavior and Mental processes of the criminal. Here the focus is “individual's criminal behavior - how it is acquired, evoked, maintained or modified”. It considers both the social and personality factors and how these are mediated by mental processes. Recently there is a shift in its focus to the cognitive aspects of offending.[3] Exploring consistent, stable personality disposition or traits was the serious study in the past.[4] In search of personality traits little attention was paid to the environment or situation. It was thought that once personality variables were identified it would be possible to determine and predict which individual was most likely to engage in criminal behavior! Search for single personality type of the murderer, rapist or psychopathic killer is not possible. ‘Criminal profiling’ refers to the process of identifying personality traits, behavioral tendencies and demographic variables of an offender based on characteristics of the crime.[5] Frequently this is based on database collected on previous offenders who have committed similar offences. Bartol *et al*[3] contend that this is 95% an art based on speculation and only 5% science. Unvalidated clinical judgement and unsubstantiated hunches are common.

## PSYCHIATRIC CRIMINOLOGY

Psychiatric criminology is also called forensic psychiatry. Traditionally, at least in America and Europe, Freudian psycho-analytic or psycho-dynamic tradition and subsequently neo-Freudian formulations are common. The psycho-analytic position assumes that one must dwell into the abysses of human personality to find unconscious determinants of human behavior, including criminal behavior. Abramsen[6] had noted “that the criminal rarely knows completely the reasons for his conduct” or as Roche,[7] emphases that ‘every criminal is such by reason of unconscious forces within him….’ In short overt behaviors are indirectly signals of symbolic dynamic, underlying attributes. Unconscious defenses distort or disguise the real meaning of absurd behavior. However, contemporary psychiatric criminology has tried to overcome traditional psychiatric criminology which had failed in replicable research data base.

## CRIMINAL BEHAVIOR: DEFINITION

The earliest definition of crime in Tappan, 1947 quoted in Bartol[3] as ‘an intentional act in violation of the criminal laws committed without defense or excuse and penalized by the state as a felony or misdemeanor’. To establish criminal behavior is that sense needs proving that it is intentional, it did not occur accidentally or without justification or excuse. To be held criminally responsible, a person must have known what he or she is doing during the criminal act and must have known that it was wrong. Surely it raises many questions.

1. Whether one should restrict oneself to a legal definition and study only those individuals who have been convicted of crime?
2. Whether one should include individuals who indulge in antisocial behavior but have not been detected by the criminal justice system?
3. Can we include persons predisposed to be criminal and how to identify them?
4. Bartol[3] reports that even by conservative estimates 16-18% of total U.S. population have arrest records for non-traffic offences (U.S Dept of justice, 1988).[8] How to include many who violate law but escape detection? Those who come to the attention but never arrested?

In U.S the system of recording incidence on basis of official police report, self-report studies and national or regional victimization studies may be fine to an extent but what about many other countries, including India where the system itself needs many corrections? Sellin[9] and others though argue about sticking to legal definition of criminal - one who is detected, arrested or committed, psychological point of view is that we should not limit ourselves to the mere legal definition. Because each society has different and changing set of values, the judgment of criminal act also varies from time to time and society to society. Many states in the U.S.A. differ significantly in their criminal codes and one continually revising them.[3] Chemical or substance possession, prostitution and pornography are classic examples of changing statue and selective enforcement.

Society and for that matter judicial system which are part of that society, perceive and process violators with some discrimination, so much so the offenders background, social status, personality, motivation, sex, age, race, legal council and circumstances surrounding the offence, all affects the legal process. It is highly likely that individuals who have been arrested, convicted or punished represent a distinctly different sample from those who participate in illegal activity but avoid detection, conviction or punishment. Also error and subjectivity cannot be removed realistically for determination of guilt, innocence or sentencing process. It is reported that if the victim is respectable citizen, the offender will receive stiffer sentence than if the case involves an ‘unrespectable’ Victim.[10] It is also reported that defendants who raped a married woman or virgin were more likely to receive longer sentences than defendants who raped a divorced woman.[11]

## CONCLUSION

The psychological or psychiatric criminology has to look beyond the individuals who have reached the final stage of the legal process to understand the ‘criminal mind’. There is a filtering as ‘suspect’, ‘arrested’, ‘charged’, ‘convicted’ to the ultimate label of convict, inmate prisoner or criminal shows funneling effect. That means only fewer and fewer individuals reach subsequent step in the criminal justice process, which is called ‘the great pyramid of legal order’ or ‘legal iceberg’.[12] Our approach should go beyond the behavior that generally qualifies as criminal. From this perspective, we can include the vast body of psychological research that deals with such areas as aggression, “deviant” sexual behavior and moral development.[3] Our focus has to be on the persistent repetitive offender, whether detected or undetected by the criminal justice system. In this process, psychiatrists are uniquely placed with a vast information which is personal, clinical and social. However, that entails higher responsibility to oneself, profession and to the society.
